# Neuroprotective Studies on *Polygonum hydropiper* L. Essential Oils Using Transgenic Animal Models

**DOI:** 10.3389/fphar.2020.580069

**Published:** 2021-01-27

**Authors:** Xin Tong, Xiaoling Li, Muhammad Ayaz, Farhat Ullah, Abdul Sadiq, Muhammad Ovais, Muhammad Shahid, Mars Khayrullin, Ali Hazrat

**Affiliations:** ^1^ Department of Clinical Medicine, Heilongjiang University of Chinese Medicine, Harbin, China; ^2^ Department of Imaging, First Affiliated Hospital, Heilongjiang University of Chinese Medicine, Harbin, China; ^3^ Department of Pharmacy, University of Malakand, Chakdara, Pakistan; ^4^ CAS Key Laboratory for Biomedical Effects of Nanomaterials and Nanosafety, CAS Center for Excellence in Nanoscience, National Center for Nanoscience and Technology, Beijing, China; ^5^ University of Chinese Academy of Sciences, Beijing, China; ^6^ Department of Pharmacy, Sarhad University of Information Technology, Peshawar, Pakistan; ^7^ K.G. Razumovsky Moscow State University of Technologies and Management (the First Cossack University), Moscow, Russia; ^8^ Department of Botany, University of Malakand, Chakdara, Pakistan

**Keywords:** *P. hydropiper*, Alzheimer’s disease, cholinesterase’s, antioxidants, aβ, essential oil

## Abstract

*Polygonum hydropiper* L. and related species are reported to possess neuroprotective potentials. In an attempt to validate its anti-Alzheimer’s potentials, leaf oils (Ph. Lo) were extensively evaluated in this study against several *in vitro* and *in vivo* models of Alzheimer’s disease. The Ph. Lo were tested against pathological targets of Alzheimer’s diseases (ADs). The *in vitro* and *in vivo* assays were done for cholinesterase inhibition, anti-radical properties and cognitive assessments using transgenic animal models. In preliminary cholinesterase inhibition assays, Ph. Lo were more active against acetylcholinesterase (AChE), butyrylcholinesterase (BChE), 1,1-diphenyl-2-picrylhydrazyl (DPPH), 2,2-azinobis (3-ethylbenzthiazoline)-6-sulfonic acid (ABTS), and hydrogen peroxide (H_2_O_2_) radicals. Subsequently, Ph. Lo was evaluated for its effects on special memory, exploratory behavior, and coordination using shallow water maze (SWM), Y-maze, open filed, and balance beam tests. Animal pre-genotyping was done *via* polymerase chain reaction (PCR) to confirm amyloid precursor protein (APP) transgene, and after completion of drug therapy, brain homogenates from the cortex and hippocampus were evaluated for cholinesterase and free radical studies. In SWM task, disease control animals treated with 10 mg/kg of Ph. Lo for 5 days exhibited significant improvement in cognitive performance indicated by low escape times on 5th day compared with normal animals. In the Y-maze test, transgenic animals showed higher spontaneous alternation behavior than disease control animals and standard control group animals. Ph. Lo therapy has improved the exploratory behavior and declined anxiety behavior in diseased animals as accessed *via* open field test. Ph. Lo administration significantly augmented the motor and coordination abilities of transgenic animals when compared to other groups of animals and declined AChE, BChE activities as well as free radicals load in the cortex and hippocampus tissues. Based on our finding, it is concluded that Ph. Lo exhibit significant neuroprotective potentials preliminary due to their anti-radicals and cholinesterase inhibitory activities. Ph. Lo need further detailed studies as potential aromatherapy against neurodegenerative disorders.

## Introduction

Alzheimer’s disease (AD), a neurodegenerative disorder of the old age, is associated with cognitive disabilities, behavioral turbulence, and imperfection in routine activities. AD is a major cause of dementia, accounting for 60–80 percent of the cases (Ali et al., 2017; Mir et al., 2019). According to AD association, about 33.9 million people of the world have been affected by AD, and its prevalence is expected to increase three folds by the next 40 years (Alzheimer’s, 2010). The pathological features of AD include the deficiency of essential neurotransmitter acetylcholine (ACh) involved in impulse transmission across the synapse, accumulation of amyloid plaques (Aβ), neurofibrillary tangles (NFTs), free radical-induced neurodegeneration, and glutametergic abnormalities (Ayaz et al., 2017a; Ovais et al., 2018). Among the behavioral tools used to assess cognitive performance, special memory, exploratory behavior, coordination and response to neuroprotective agents, shallow water maze (SWM), Y-maze, open field, and balance beam are frequently applied (Eisdorfer, 2013). SWM, which is a modified version of Morris water maze (MWM), is specifically designed for mice as they feel difficulty in swimming during MWM tests. This is paddling maze utilizing shallow water which stimulates animals for escape. After drug therapy, escape latency is recorded for experimental animals. SWM is an effective tool for the assessment of special memory in rodents preferably mice (Deacon, 2013). Y-maze is another behavioral tool widely used to assess special memory in the form of spontaneous alternation behavior (Pocivavsek et al., 2016). Open field and balance beam tests are used to measure exploratory behavior and motor coordination in animals, respectively (Gould et al., 2009; Luong et al., 2011).

Currently, five drugs (galantamine, tacrine, rivastigmine, donepezil, and memantine) are clinically approved for the management of AD (Ayaz et al., 2015; Sadiq et al., 2015; Imran et al., 2017). These drugs have limited efficacy and are associated with anti-cholinergic side effects. Consequently, there is an imperative need to search out potential anti-Alzheimer’s agents with better efficacy and safety profiles. At present, there has been growing interest to investigate natural products that could play a vital role in novel drug discovery owing to the diverse nature of biocompatible secondary metabolites in plants (Rates, 2001). Various researchers have isolated numerous compounds from medicinal plants that possess significant neuroprotective and anti-Alzheimer’s capacities (Hiremathad, 2017; Ayaz et al., 2017b; Sadiq et al., 2018). Among the available drugs, galantamine is derived from plant sources, whereas rivastigmine is a synthetic derivative of naturally occurring compound physostigmine (Ullah et al., 2016). Compared to the randomly synthesized compounds, the success rate in developing drugs from medicinal plants is considerably high and this fact signifies the search of novel drugs from medicinal plants (Koehn and Carter, 2005). Several compounds from medicinal plants are reported to exhibit efficacy on multiple targets of AD. For instance, curcumin modulates inflammatory pathways in the CNS, prevents Aβ aggregation, and scavenges free radicals (Frautschy et al., 2001; Lim et al., 2001; Baum and Ng, 2004; Ono et al., 2004; Yang et al., 2005). Consequently, it is in clinical trials as a potential multi-active agent against AD. Several other active phytoconstituents including catechins, myricetin, and gossypetin also inhibit enzymes, Aβ aggregation, and scavenge free radicals (Rice-Evans et al., 1996; Ayaz et al., 2019).

*Polygonum hydropiper* L., family Polygonacae, is used in folk medicine for the treatment of various inflammatory diseases caused by free radicals (Ayaz et al., 2014; Huq et al., 2014; Mihaylova et al., 2019; Ayaz et al., 2020). Additionally, other species of the family have been explored for their potential therapeutic agents against AD and dementia (Chan et al., 2003; Li et al., 2005; Chen et al., 2007). We recently reported the efficacy of β-sitosterol isolated from *P. hydropiper* against oxidative stress, cholinesterase enzymes, and cognitive dysfunctions using the transgenic animal model (Ayaz et al., 2017a). In another preliminary study, we investigated the *in vitro* cholinesterase inhibitory and antioxidant potentials of essential oils from leaves and flowers of *P. hydropiper* (Ayaz et al., 2015). Based on *in vitro* results, the current study was designed to appraise Ph. Lo for its efficacy against cognitive dysfunctions in the transgenic animal model of AD.

## Materials and Methods

### Plant Collection, Processing, and Isolation

The whole plant of *P. hydropiper* was collected in July 2013, authenticated by Dr Gul Rahim, a botanical taxonomist and in charge of the University of Malakand herbarium. Plant dried sample was placed at the university herbarium, with voucher No. H.UOM.BG.107. Whereas for extraction of leaf oils (Ph. Lo), leaves were collected in September 2014 from Talash Dir. Leaves were properly cleansed with distilled water to remove any adulterants. Ph. Lo were collected *via* Clevenger apparatus and subjected to GC–MS analysis for the identification of major constituents as reported previously (Ayaz et al., 2015).

### Animals Used and Ethical Approval

Based on our preliminary *in vitro* results (Ayaz et al., 2015), Ph. Lo were subjected to detailed *in vivo* studies using double transgenic animals. Transgenic animals expressed with human APP weighing 35–40 g were used in the study (Clark, 2011). This study was evaluated and approved by Departmental Research Ethics Committee (DREC) with reference no. DREC/20160502/01. All animals were maintained under standard conditions and provided food and water *ad libitum*.

### Genotyping

**FIGURE 1:**
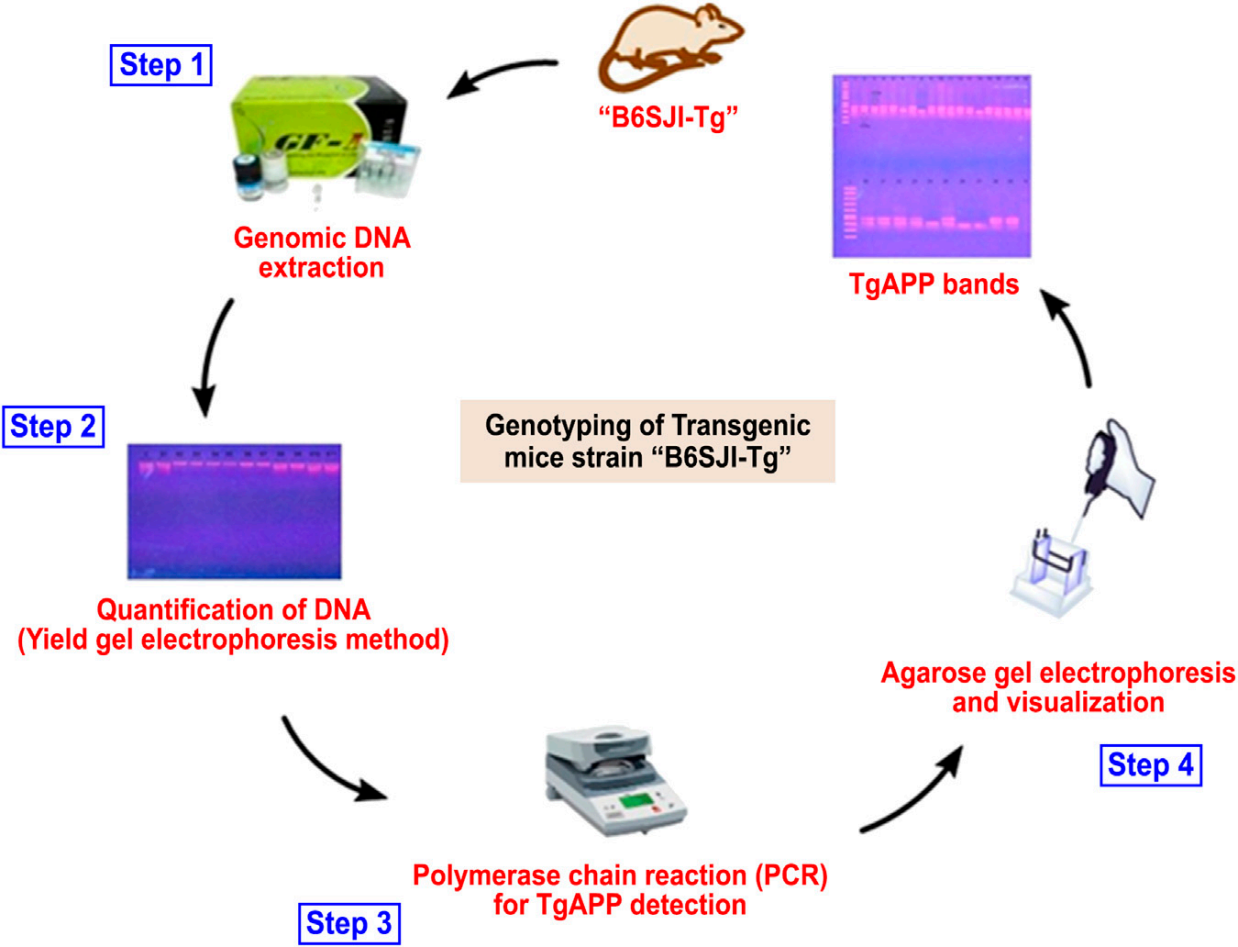
Overview of the genotyping process for the identification of transgenic animals.

Animals were subjected to genotyping *via* PCR for confirmation of transgene before detailed studies following our previously reported protocol (Ayaz et al., 2017a) as summarized in Table 1, Figure 1.

**TABLE 1:** Primers sequences for PCR reaction.

Primer	Sequence 5’→3’	Primer type
IMR3610	AGG ACT GAC CAC TCG ACC AG	Transgene
IMR3611	CGG GGG TCT AGT TCT GAC T	Transgene
IMR7338	CTA GGC CAC AGA ATT GAA AGA TCT	Internal positive (F)
IMR7339	GTA GGT GGA AAT TCT AGC ATC ATC C	Internal positive (R)

### Animal Grouping and Behavioral Studies

Animals confirmed with transgene were grouped into four, each group containing eight animals (*n* = 8); disease control (untreated transgenic animals provided with normal diet but no drug therapy), standard control (transgenic animals provided with reference drug galantamine), test control (transgenic animals provided with Ph. Lo), and additional normal control group (normal animals on usual diet and no drugs).

### Administration of Drugs

The disease control group containing transgenic animals maintained on normal diet, test control group containing transgenic animals administered with Ph. Lo at 10 mg/kg body weight/day (I/P), and reference group transgenic animals received galantamine at the same dose for consecutive 5 days. In the normal control group, normal animals were trained and maintained on the standard diet without any drug administration.

### Shallow Water Maze (SWM) Task

Shallow water maze (SWM) was designed (MK2 model/octagonal) following published procedure in the journal of visual experiments (JOVE) (Deacon), and it was already reported in our previous work (Ayaz et al., 2017a). Shallow water (20–25°C) was maintained in the maze which provides sufficient escape stress to the experimental animals. Maze contains eight true–false exits, among which seven are clogged whereas one is open with 40-mm diameter for smooth escape.

#### Pre-training

Experimental animals were divided in different groups, properly marked on the tails for identification, and were subjected to training trials. Each animal received three trails a day, whereby, animals were placed opposite to exit hole and allowed to escape. Subsequent to the end of maximum time (60 s) if animal failed to find the exit, it was manually guided toward the exit way. Each trial was 1 h apart and continued for 9 days.

### Y-maze Task

Y-maze is another behavioral tool used for the cognitive assessments of rodents. It consists of three arms, each 30 × 8 × 20 cm having two false and one true exit. Shallow water of about 2 cm depth maintains an escape stress, and visual aids help the animals to develop memory and remember the true exit (Ayaz et al., 2017a).

#### Pre-training and Test Trials

All experimental animals were trained for nine consecutive days, with three trials per day (each trial 1 h apart). To start a trial, animals were placed randomly at point opposite to the true exit and allowed to find escape for 60 s. Moreover, percent spontaneous alternation behavior was calculated as

$$\text{\% Alteration behavior}=\frac{\text{Successive triplet sets}}{\text{Number of Total enteries}}-2\times 100,$$

At the end of training trials, each animal of the test control group received Ph. Lo at 10 mg/kg body weight (I/P) for 5 days. Whereas the standard control group was administered galantamine, and both parameters were recorded.

### Open Field

Open field is an extensively used behavioral tool for the assessment of locomotor behavior of the animals (Arendash et al., 2001). The experimental tool consists of open box usually of 81 × 81-cm dimensions and 28.5-cm walls. The box floor is usually colored with four lines on each side to make 16 squares of 20 × 20 cm. During test and training trials, animals were introduced into the center of the box and permitted to search for 5 min. Average number of line crossed by animals was noted. Moreover, animals were allowed in the apparatus for 30 min, and time spent in the central and peripheral areas was recorded (Choleris et al., 2001).

### Balance Beam

Subsequent to 9 days training, animals were administered with standard and test drugs (I/P) at the above doses and allowed to cross the wooden beam. Times (sec) were recorded for the animals to successfully cross the beam without slips and discoordination.

### Neurochemical Studies

#### Cholinesterase Inhibitory Studies on Cortical and Hippocampus Brain Tissues

Owing to the significance of ACh in neurotransmission and cognition, activity of ACh inactivating enzymes (AChE and BChE) was analyzed in the brain tissue homogenates (Whitehouse et al., 1982). On the last day (5th day) of drug therapy, 1 h of postdrug administration, animals were sacrificed, and cortical and hippocampus tissues were dissected out in ice cold phosphate buffer following our previously reported procedure (Ayaz et al., 2017a). Brain tissues were isolated in 0.1 M phosphate buffer saline with 8.0 pH and were homogenized. Thereafter, the homogenates were centrifuged at 1,000 × *g* for about 10 min, and supernatant part was collected. Protein content of the supernatants was estimated *via* the Bradford method (Bradford, 1976; Isomae et al., 2002) and was used as an enzyme source following already established Ellman’s assay (Ellman et al., 1961; Trevisan et al., 2003).

#### Assessments of FC and HC Free Radicals Load

Keeping in view the role of free radicals in neurodegenerative disorders, free radical load was assessed in cortical and hippocampus tissue homogenates post–drug therapy following the procedure previously reported (Ayaz et al., 2017a). Frontal cortex (FC) and hippocampus (HC) tissue homogenates corresponding to 0.1 mg/ml proteins were homogenized with 1 ml methanol and mixed with 0.4 ml previously prepared DPPH solution (0.1 mM). Pure DPPH solution was used as control, and antioxidant activity was checked using formula

$$\text{DPHH inhibition }\left(\text{\%}\right)\;=\;\frac{\text{Abosrbance of control}-\text{absorbance of sample}}{\text{absorbance of control}}\times \;100,$$

### Statistical Analysis

All experiments were performed in triplicate, and results were expressed as mean ± SEM. In SWM tests, two-way repeated measures ANOVA followed by *post hoc* Bonferroni’s tests were used. For Y-maze and brain tissue studies, one-way ANOVA followed by *post hoc* Tukey’s test was employed. A value of *p* < 0.05 was considered as statistically significant. Whereas statistical analyses and figures were constructed *via* Graph Pad Prism-5 (Graph Pad Software Inc., San Diego, CA, United States).

## Results and Discussion

AD is a neurodegenerative disorder of the aging brain that is affecting large population, while its prevalence is expected to increase tremendously by 2050. The major issues associated with AD are the cognitive decline and behavioral turbulence which adversely affect the routine life of patients. Consequently, there is an immense need to develop more cost-effective drugs for the treatment of AD. In this scenario, natural products including medicinal plants provide an alternative platform for the development of novel therapeutic agents against AD. These phytotherapetic agents are cost-effective and biocompatible as compared to randomly synthesized compounds.

In our previous study, we reported in detail the phytochemistry and significance of identified compounds as potential AChE/BChE inhibitors (Ayaz et al., 2015). Among these, limonene is reported to provide neuroprotection against Aβ_42_-induced neurotoxicity in animal models of AD (Shin et al., 2020). Likewise, β-elemene enhances locomoter activity in animal models by reducing oxidative stress, neuronal apoptosis (Wang et al., 2018), and reduces inflammation in traumatic brain injury (Meng et al., 2018). β-caryophyllene offers numerous neuroprotective properties and reduces neuroinflammation (Francomano et al., 2019). Several other compounds like nerolidol (Javed et al., 2016), benzofuran (Cho et al., 2015), farnesene (Turkez et al., 2014), β-mycrene (Ciftci et al., 2014), juniper camphor (Bais et al., 2015), and androstan (Cai et al., 2017) have well established antioxidant, anti-neuroinflammatory, and neuroprotective potentials as reported in these studies. These components might contribute to the overall neuroprotective properties of Ph. Lo. Subsequently, the Ph. Lo being more active were evaluated in more detail using transgenic animal models.

### Shallow Water Maze (SWM) Paradigm

**FIGURE 2:**
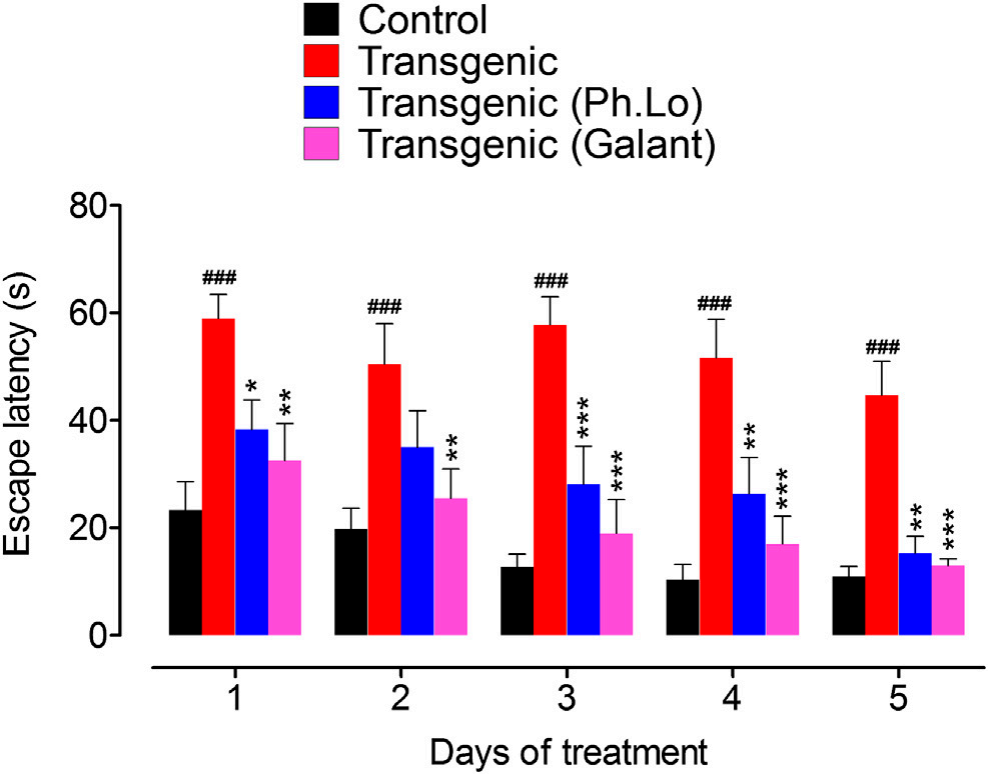
Results of the SWM test performed on various groups of animals. Each value represents an average ± SEM of three independent experiments. **, ##: *p* < 0.01; ***, ###: *p* s < 0.001. ns: values significantly different as compared to the normal group.

Keeping in view the ethno-pharmacological profile and our preliminary studies, the Ph. Lo were studied in detail using the transgenic animal model of AD. SWM is a recently developed tool and adopted from Morris water maze (MWM) as some rodents feel trouble in swimming. Both tools are specialized and extensively employed in behavioral neuroscience for the cognitive assessment (Morris, 1981). In the SWM test, disease control animals treated with 10 mg/kg of Ph. Lo for 5 days showed significant improvement in cognitive performance indicated by low escape times **(** Figure 2). As observed on day 1, a gradual improvement was observed in transgenic animals with a steady decline in escape time as noticed from day 1 38.33 ± 7.76–15.33 ± 4.39 s on 5th day of treatment, which was found to be comparable with the normal animals. Transgenic untreated animals showed high escape time (59.00 ± 6.28 s day one) with no significant change (44.66 ± 8.96 s on 5th day) during the training and test trials. In contrary, transgenic animals treated with standard drug galantamine exhibited gradual improvement in cognitive performance with gradual decline in escape times in comparison to test control and disease control animals. Essential oil from *Zataria multiflora* also improves learning and special memory in rats using Morris water maze task (Eskandari-Roozbahani et al., 2019). Cardamom oil supplementation for 42 days significantly reduced (*p* < 0.001 in comparison to diseases control group) escape latency in aluminum chloride–administered animals, which signify their cognitive enhancing potentials (Auti and Kulkarni, 2019). Further, it also reduced aluminum chloride induced increase in transfer latency in elevated plus maze. Carvacrol, an essential component of volatile oils, rescued learning and memory dysfunctions in reperfusion-induced ischemic model using the Morris water maze model (Guan et al., 2019). Likewise, Antarctic Krill oil improved special learning and memory in SAMP8 mice using Morris water maze and Barnes maze indicated by decline escape latency (Li et al., 2018).

### Y-maze Paradigm

**FIGURE 3:**
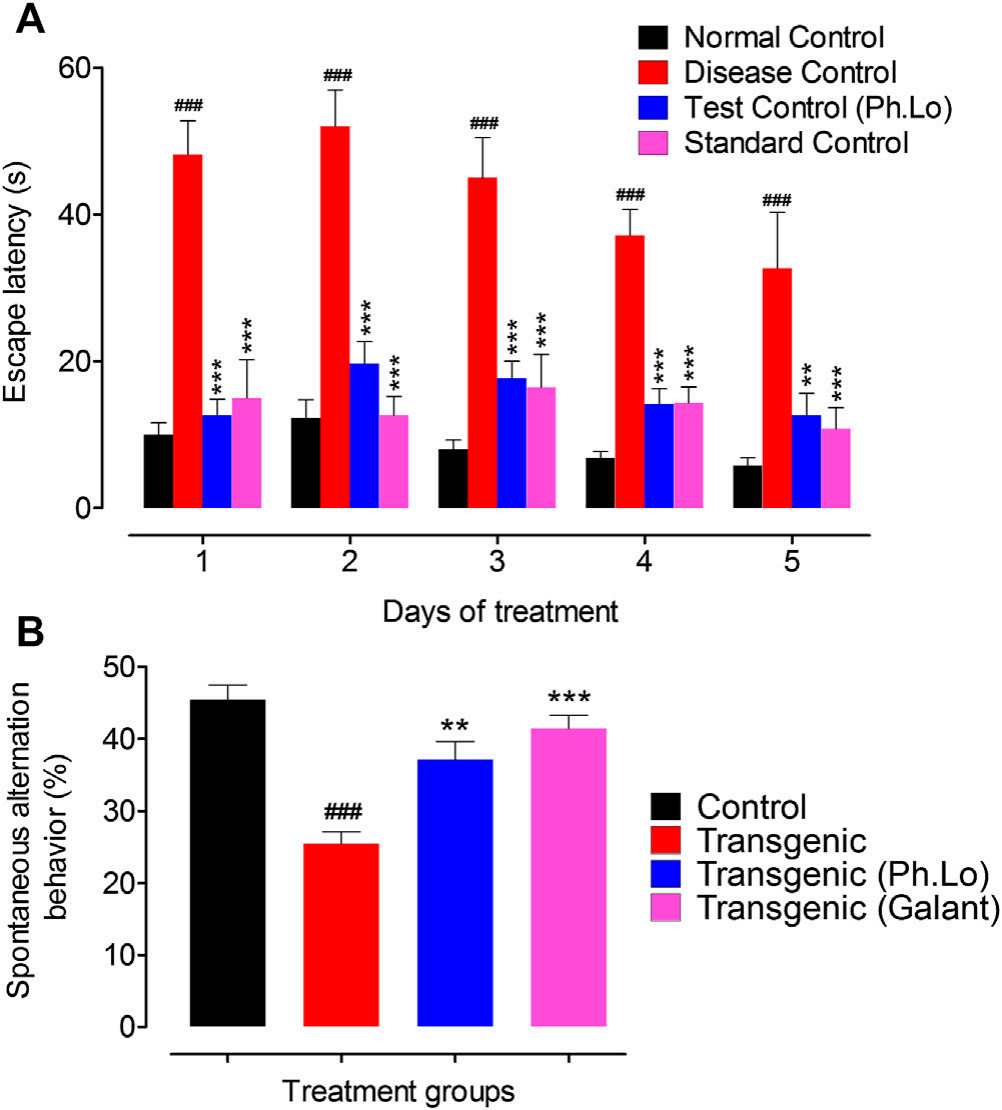
Y-maze test on various groups of animals. **(A)** Escape latency (sec). **(B)** Spontaneous alternation behavior (%). Each value represents an average ± SEM of three independent experiments. **, ##: *p* < 0.01; ***, ###: *p* s < 0.001.

Animals were also tested using another cognitive assessment tool Y-maze. Transgenic animals treated with 10 mg/kg of Ph. Lo showed considerable decline in escape time during the course of therapy and improved spontaneous alternation behavior (Figures 3A,B). The average escape times observed were 12.75 ± 3.04 (day 1st), 19.66 ± 4.22 (day 2nd), 17.66 ± 3.25 (day 3rd), 14.16 ± 2.93 (day 4th), and 12.50 ± 4.33 s on 5th day of Ph. Lo administration. Spontaneous alternation was much improved 37.33 ± 4.17% in transgenic animals on 5th day of Ph. Lo therapy in comparison to disease control animals (26.33 ± 2.38%) and standard control group animals (40.83 ± 2.99), respectively. Previously, several essential oils and their components were reported to improve cognitive performance of animals indicated by improved spontaneous alternation activity. For instance, α-pinene, a volatile component, is reported to considerably improve special recognition memory in scopolamine-induced amnesia model using Y-maze (Lee et al., 2017). Oils from other sources like fish oil and peanut oils mixture are reported to improve spontaneous alternation behavior in the Y-maze test (Xiang et al., 2016).

### Open Field Study

**FIGURE 4:**
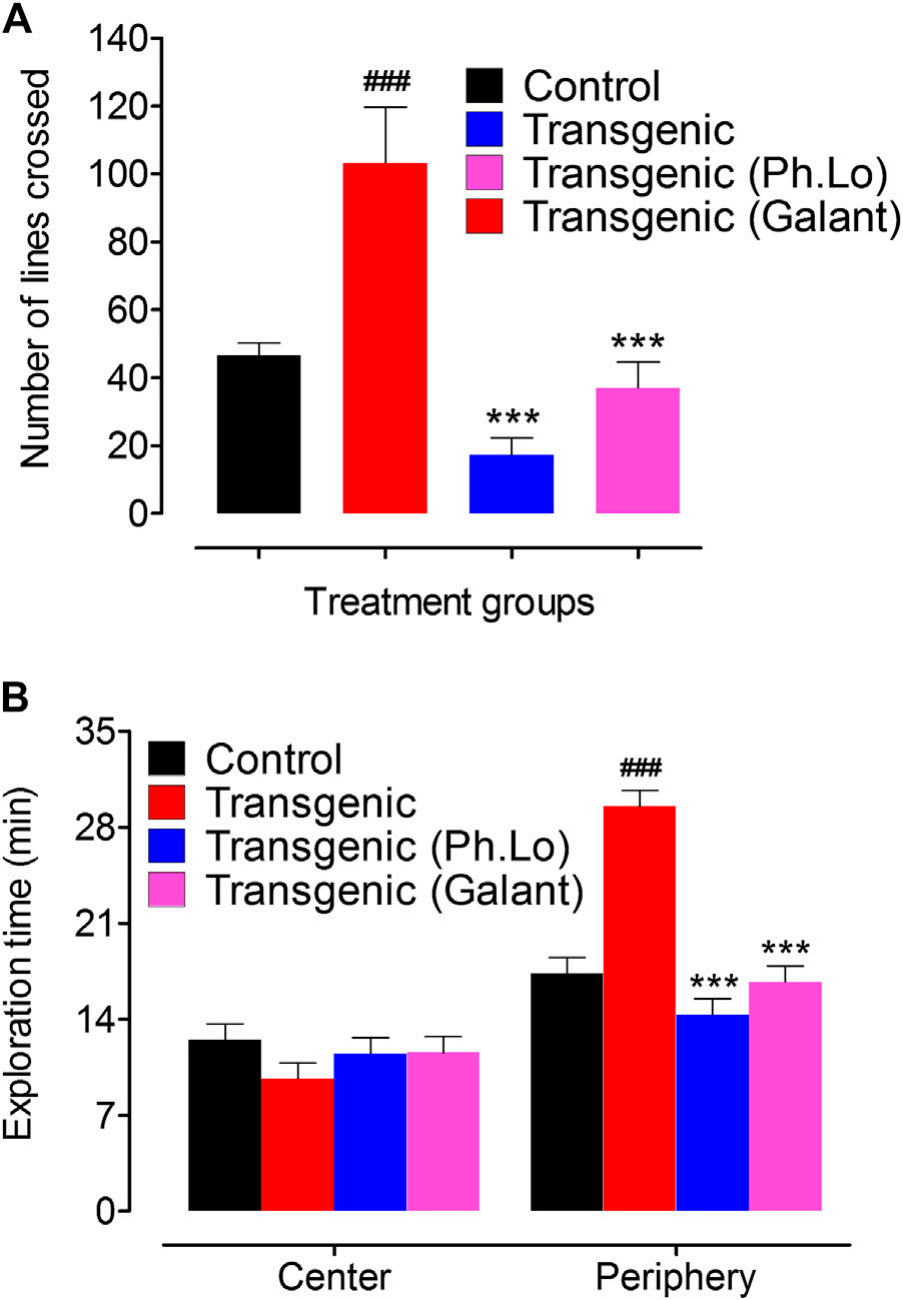
Effect of Ph. Lo in the open field test in various groups of animals. **(A)** Number of lines crossed by various group of animals. **(B)** Exploration times in minutes.

Pharmacological effect of Ph. Lo on the exploratory behavior (locomotion) and anxiety was assessed *via* the open field test. Transgenic animals (untreated) showed less exploratory behavior indicated by number of line crossing (11.00 ± 0.57) and spent 9.66 ± 0.33 and 29.50 ± 1.72 min in central and peripheral chambers of the compartment (Figures 4A,B). The same animals given 10 mg/kg Ph. Lo exhibited improvement in exploratory behavior (46.00 ± 5.19 lines crossed in 30 s) and spent 14.33 ± 0.88 and 11.50 ± 0.86 min in peripheral and central areas indicating less anxiety, when compared to the diazepam-treated group. The Ph. Lo therapy was comparatively more effective than our previously reported work on beta-sitosterol (Ayaz et al., 2017a), possibly *via* the liphophilic character of Ph. Lo to cross BBB (Agatonovic-Kustrin et al., 2020). Effect of *Eplingiella fruticosa* leaf essential oil on locomotor behavior of animals is previously reported (Beserra-Filho et al., 2019). Their results revealed a significant improvement in locomotor activity of the animals. Antarctic krill oil also improves special memory, exploratory, and locomoter activity of SAMP8 mice using open field as indicated by increased movements and reduced anxiety (Li et al., 2018). Cardamom oil administered for 42 days at 100 and 200 mg/kg orally has significantly improved (*p* < 0.001) locomotor behavior of rats in the aluminum chloride–induced neurotoxicity model (Auti and Kulkarni, 2019).

### Balance Beam Study

**FIGURE 5:**
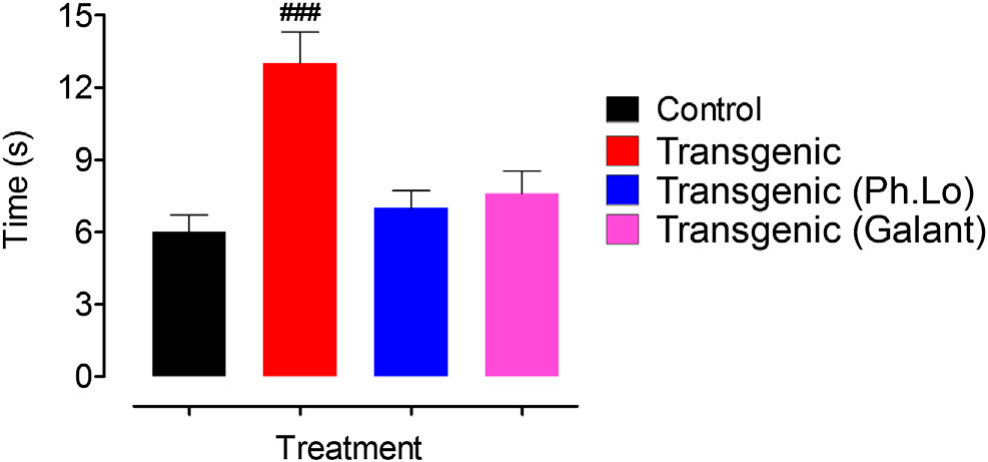
Effect of Ph. Lo on motor skills and coordination in animals groups.

In order to appraise the effect of Ph. Lo on the balance and coordination of transgenic animals, the balance beam test was used (Luong et al., 2011). This tool is widely used to analyze motor skills in rodents and response to drug therapy after brain injuries and diseases. Ph. Lo administration significantly augmented the motor and coordination abilities of transgenic animals when compared to other groups of animals **(** Figure 5). Tyrosol, a phenolic component of olive oil, exhibited neuroprotective potentials in the cerebral ischemic model. Tyrosol at dose of 10, 20, and 30 mg/kg significantly improved balance and coordination in animals as indicated by latency time (Bu et al., 2007). Likewise, β-caryophyllene, which is identified in Ph. Lo and so many other essential oils, is reported to improve motor coordination in the MPTP-induced Parkinson’s diseases model using balance beam paradigm (Bu et al., 2007).

### Neurochemical Studies

#### Effect of Ph. Lo on Cortical and Hippocampus AChE

**FIGURE 6:**
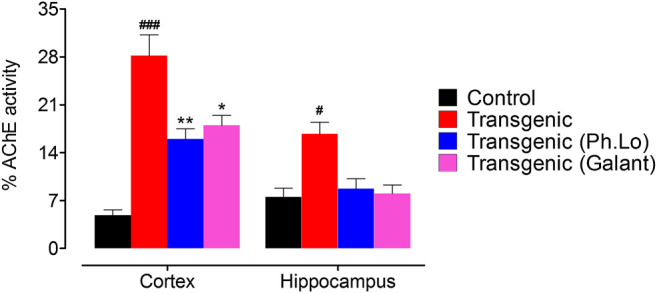
AChE activity in the cortex and hippocampus tissues.

It is now well established that deterioration of cholinergeic neurons (ACh releasing neurons) whose cell bodies are present in the basal forebrain occur in AD patients. As these neuron innervate cortex and other areas, as a result of their gradual degeneration cognitive decline and memory impairment results (Coyle et al., 1983). Thus, the brain cholinergic system is targeted as potential treatment for AD patients (Giovannini et al., 1997). One strategy is the use of ACh degrading enzymes to maintain it for prolong time at the synapse and to improve its pharmacological activity (Oddo and LaFerla, 2006; Martorana et al., 2010). Keeping in view the significance of ACh in neurotransmission, cognition, and its deficiency in AD patients (Whitehouse et al., 1982), the AChE level was analyzed in the FC and HC areas of the brain. In the current study, we observed that the activity of ACh degrading enzymes was considerably declined which subsequently improved the cognitive performance of experimental animals. AChE activity was considerably low in FC (15.66 ± 2.02%) and HC (8.66 ± 2.02%) tissue homogenates in comparison to the standard group animals (Figure 6).

#### Effect of Ph. Lo on Cortical and Hippocampus BChE

**FIGURE 7:**
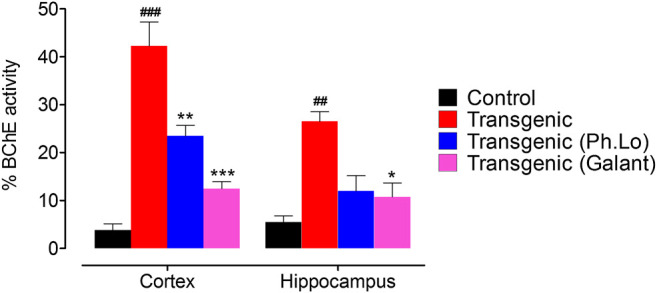
BChE activity in the cortex and hippocampus tissues.

ACh plays a significant role in cholinergic signaling in the brain which results in transmission of impulses across the synapse. BChE beside AChE hydrolyzes and recycles ACh after release in the synapse, especially when the AChE level is down. So both AChE and BChE are potential targets to inhibit ACh degradation and relieve symptomology of the AD patients. BChE levels are reported to increase in AD patients and their inhibition *via* the use of cholinesterase inhibitors can restore the level of ACh at synapse and enhance cognitive performance of AD patients (Nordberg et al., 2013; Jasiecki and Wasąg, 2019). In the current study, considerable BChE activity was observed in the FC (44.00 ± 6.65%) and HC (24.66 ± 1.33%) area of the brain in the disease group of animals (Figure 7). The BChE activity was significantly reduced by the galantamine as recorded for the standard control group in the FC and HC regions of transgenic animals having percent BChE activity of 12.33 ± 3.48 (*p* < 0.05) and 12.66 ± 2.02 (*p* < 0.001), respectively. Moreover, the BChE activity was observed as 22.00 ± 1.88% in FC of transgenic animals treated with Ph. Lo which was recorded to be significantly different (*p* < 0.01) as compared to the disease control group. The Ph. Lo treated group of transgenic animals showed BChE activity 14.00 ± 2.82% with *p* > 0.05. Several clinical trials with aromatherapy are reported to improve cognitive performance, reduce agitation, aggression, and psychotic symptoms in AD and Parkinsonism patients (Jimbo et al., 2009; Ali et al., 2015; Scuteri et al., 2017). However, the exact underlying mechanisms of these beneficial effects are not fully discovered yet.

#### Effect of Ph. Lo on Cortical and Hippocampus Free Radicals

**FIGURE 8:**
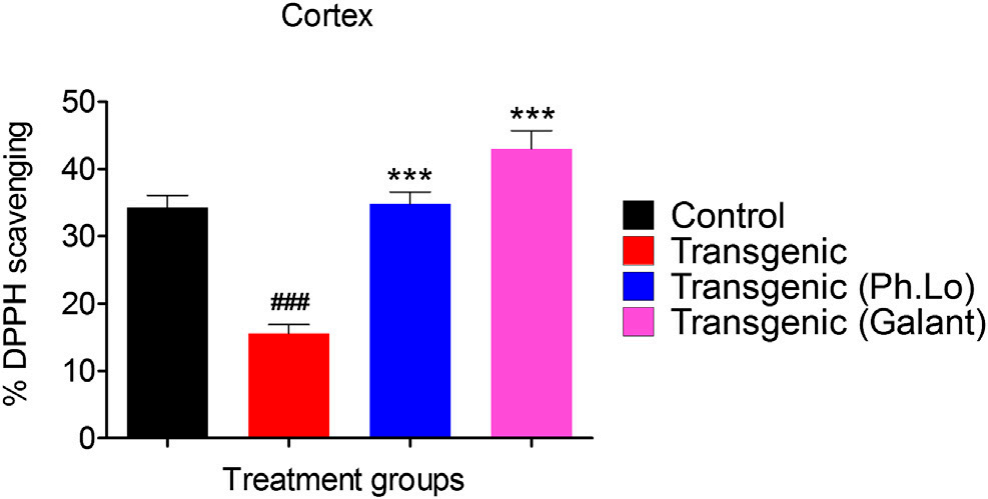
Effect of Ph. Lo therapy on free radicals in FC.

**FIGURE 9:**
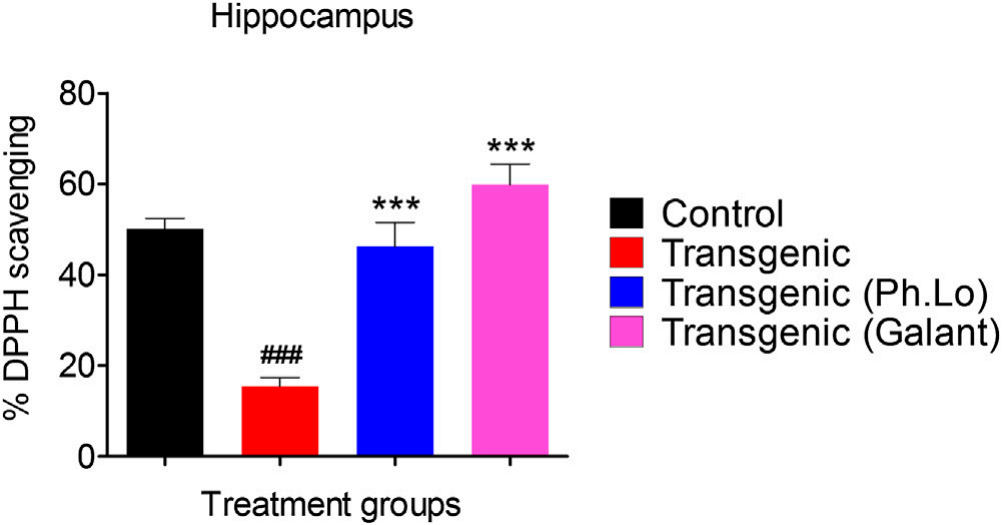
Effect of Ph. Lo therapy on free radicals in HC.

Results of the DPPH free radical scavenging assay (*ex vivo*) in the cortex have been summarized in Figures 8 and 9. The free radical scavenging potential recorded for the standard control group, that is, ascorbic acid was 43.33 ± 4.66%. The antioxidant potential shown by the disease control group was recorded to be 15.33 ± 2.02%. Whereas the Ph. Lo treated group exhibited antioxidant potential of 32.33 ± 1.45% in the cortical tissues of transgenic animals. Moreover, the ascorbic acid–treated group and transgenic animal groups treated with Ph. Lo demonstrated 57.66 ± 6.43% and 52.33 ± 6.43% antioxidant effects, respectively. Previous studies indicated that cardamom oil administration reduced free radical load in the cortex and hippocampus and improved antioxidant enzymes in the aluminum oxide–induced neurotoxicity model (Auti and Kulkarni, 2019). The present study demostrated that Ph. Lo have significant *in vitro* cholinesterase inhibitory and free radical scavenging potentials. Moreover, AChE and BChE inhibitory activities in the FC and HC tissues of transgenic animals were also demostrated, which suggest its ability to inhibit these target enzymes in the brain regions. Hence, ACh level is maintained for prolong time in synaptic cleft which stimulates the cholinergic receptors. Ph. Lo essential oils–mediated enhanced cholinergic transmission can be useful for memory restoration in AD and also preventing free radical–induced neurodegeneration in the aging brain.

## Conclusion

Our previous anti-cholinesterase and antioxidant studies on Ph. Lo and Ph. Fo motivated us for further detailed studies in specific animal models. In this study, Ph. Lo exhibited significant improvement in cognitive performance of the animals in SWM, Y-maze, open field, and balance beam studies. Moreover, Frontal cortex and hippocampus studies of the treated transgenic animals revealed that Ph. Lo significantly inhibit cholinesterase's in the target sites and reduce free radical load. However, further studies regarding their exact mechanism of absorption into their target sites inside the brain are required. The combined antioxidant and cholinesterase inhibitory potentials of the oils indicate that it is effective on more than one target which advantageous as compared to currently available drugs. We suggest further studies regarding its effects on Aβ.

## Data Availability Statement

The raw data supporting the conclusions of this article will be made available by the authors, without undue reservation.

## Ethics Statement

The animal study was reviewed and approved by the Departmental Research Ethics Committee (DREC) with reference no. DREC/20160502/01.

## Author Contributions

MA, FU, AS, MS, MO, and AH performed experimental, data analysis, and drafting of original manuscript. XT, XL, and MK refined the manuscript, funded the project, and helped in revising the manuscript after review. All authors read and approved the final version of the manuscript.

## Conflict of Interest

The authors declare that the research was conducted in the absence of any commercial or financial relationships that could be construed as a potential conflict of interest.
